# Oxytocin enhances oligodendrocyte development and improves social deficits in autistic rats

**DOI:** 10.3389/fnins.2025.1624932

**Published:** 2025-08-12

**Authors:** Min Wen, Shuang Zheng, Hongbo Luo, Yi Zhang, Bo Zhou

**Affiliations:** ^1^State Key Laboratory of Discovery and Utilization of Functional Components in Traditional Chinese Medicine, Guizhou Medical University, Guiyang, Guizhou, China; ^2^College of Pharmacy, Guizhou Medical University, Guiyang, Guizhou, China; ^3^College of Basic Medical, Guizhou Medical University, Guiyang, Guizhou, China; ^4^Guizhou Provincial Engineering Technology Research Center for Chemical Drug R&D, Guizhou Medical University, Guiyang, Guizhou, China; ^5^Department of Pediatric Healthcare, Qianxinan People's Hospital, Xingyi, Guizhou, China

**Keywords:** autism spectrum disorder, oxytocin, amygdala, oligodendrocyte, mitochondrial, PI3K/AKT pathway

## Abstract

**Purpose:**

Autism spectrum disorder (ASD) is a neurodevelopmental condition with complex etiological factors, including genetic predisposition and environmental influences. In particular, exposure to environmental stressors in utero has increasingly been implicated in disrupting fetal neurodevelopment and potentially contributing to the pathogenesis of ASD in offspring. The aim of this study was to investigate the therapeutic potential of oxytocin and to elucidate its underlying molecular mechanisms in a valproic acid (VPA) exposure-induced rat model of ASD.

**Methods:**

To generate the ASD offspring model, pregnant rats received intraperitoneal injections of VPA on embryonic day 12.5 (E12.5). A control group was administered saline instead. Only male offspring were included in subsequent experiments. On postnatal day 21 (P21), VPA-exposed offspring were randomly divided into: (1) VPA group (ASD model) and (2) VPA+OT (oxytocin inhaled daily, 400 ug/kg, P21-42) group. Behavioral assessments (social behaviors, stereotyped behaviors, anxiety-like behaviors) and amygdala RNA sequencing were compared across control group, VPA group, and VPA+OT group. Both threshold and threshold-free bioinformatics analysis methods were employed to identify the potential therapeutic mechanisms of oxytocin. The findings were further validated using transmission electron microscopy and qPCR.

**Results:**

Intranasal oxytocin administration significantly ameliorated social deficits, repetitive behaviors, and anxiety-like responses in ASD model rats. Transcriptomic profiling revealed substantial neurodevelopmental abnormalities in VPA group. Consistent results from GSEA enrichment analysis, dynamic gene expression pattern analysis and WGCNA showed significant suppression of oligodendrocyte development and differentiation in the VPA group. Pathway analysis indicated that this functional inhibition was associated with the PI3K/AKT signaling pathway. Oxytocin may promote oligodendrocyte development and differentiation by activating the PI3K/AKT pathway, thereby ameliorating social deficits. Further validation by transmission electron microscopy and qPCR confirmed that oxytocin treatment improved myelination deficits in the ASD rat model.

**Conclusions:**

Our findings demonstrate that oxytocin significantly improve social interaction deficits in the VPA-induced autism model, which may be related to its activation of the PI3K/AKT pathway to promote oligodendrocyte development and differentiation.

## 1 Introduction

Autism is a group of neurodevelopmental disorders with highly heterogeneous clinical manifestations that severely impair language formation, emotional cognition and social behavior in children. It leaving most affected children unable to live independently and in need of lifelong care, making it one of the most devastating childhood diseases for families, society and affected children (Tafolla et al., 2025; Lord et al., 2018; Hirota and King, 2023). At present, the precise molecular mechanisms of ASD are still not fully elucidated and no drug on the market are currently approved for ameliorating the core symptoms of autism (Baribeau et al., 2022).

Oxytocin, a neuropeptide hormone synthesized in the paraventricular and supraoptic nuclei of the hypothalamus and subsequently secreted by the posterior pituitary gland, plays a crucial role in obstetrical practice, particularly in facilitating uterine contractions during childbirth and preventing postpartum hemorrhage (Uvnäs-Moberg, 2024; Hermesch et al., 2024). Beyond its peripheral functions, a growing body of neuroscientific evidence from human (Zink, 2012; Heinrichs, 2008; Bos et al., 2012; Striepens et al., 2011) and *in vivo* experiments on mammals (Froemke and Young, 2021; Josselsohn et al., 2024; Marsh et al., 2021; Resendez et al., 2020; Menon and Neumann, 2023) suggests oxytocin as a pivotal neuromodulator in the regulation of complex social behaviors, including social cognition, emotional bonding, and social behavioral responses, leading to its recognition as the “prosocial neuropeptide” in modern neuroendocrinology research. Emerging evidence from recent years has increasingly implicated oxytocin as a critical modulator in the pathogenesis and potential treatment of neurodevelopmental disorders, particularly ASD. Szabo et al. (Szabó et al., 2024) demonstrated that chronic intranasal oxytocin exposure can reliably ameliorate social deficits in the Shank3B^−/−^ mouse model of autism as early as after 2 weeks of administration. (Choe et al. 2022) show that Cntnap2-deficient mice, a well-established genetic model of ASD, exhibit significant disruptions in functional brain connectivity patterns. Their research also revealed that systemic administration of exogenous oxytocin not only restores normal neural connectivity but also ameliorates core social behavioral deficits in these transgenic mice. (Bertoni et al. 2021) found peripheral oxytocin administration in neonates can potentially restore hippocampal neural circuitry and significantly ameliorate social behavioral impairments in a mouse model of autism. Despite promising preclinical evidence supporting oxytocin's therapeutic potential for ASD, its translation into clinical practice faces substantial challenges. Current evidence indicates that clinical trial outcomes show significant variability, with the efficacy of oxytocin interventions being strongly influenced by both individual patient characteristics (including genetic polymorphisms, developmental stages, sex differences, and clinical subtypes) and intervention parameters (such as optimal dosage, administration routes, and treatment duration) (Kiani et al., 2023; Hu et al., 2023). Given these inconsistencies in clinical efficacy, there is an urgent need to explore the molecular basis and neural mechanisms of its regulation of social deficits in animal experiments, in order to provide a robust scientific foundation for optimizing its clinical application in ASD treatment.

Oligodendrocytes (OL) are the central nervous system (CNS) cells that generate the multilayered myelin membrane sheath surrounds axons of vertebrate neurons, providing metabolic support to neurons and having an important role in maintaining axonal integrity and energy metabolism (Cherchi et al., 2021). Deficits in Oligodendrocyte development and function will lead to demyelination and axonal dysregulation, and disruption in neuron–glia interactions promote autistic-like features (Bronzuoli et al., 2018; Duncan et al., 2021; Galvez-Contreras et al., 2020; Takanezawa et al., 2021). Pharmacological facilitation of myelin formation has been shown to improve social deficits in autistic model (Bohlen et al., 2023; Bao et al., 2024; Gong et al., 2025). Emerging evidence indicates that oxytocin plays a significant role in modulating oligodendrocyte development, including their proliferation, differentiation, and subsequent myelination processes in the CNS (Havránek et al., 2017; Mairesse et al., 2019; Dalton et al., 2024). Based on this evidence, we propose that oxytocin may improve ASD symptoms by regulating oligodendrocyte development and myelination, thereby restoring white matter connectivity and optimizing neural network function.

Valproate sodium (VPA), a commonly prescribed antiepileptic drug, has been definitively shown to significantly increase ASD incidence in offspring when administered during early pregnancy, and is consequently widely used to generate ASD rat models (Nicolini and Fahnestock, 2018; Smith and Brown, 2013; Tartaglione et al., 2019). The model recapitulates the core features of human ASD, including social communication deficits and restricted/repetitive behaviors, while providing insights into the pathophysiology to neurodevelopmental disorders. It is widely used for therapeutic evaluation and molecular mechanism studies of ASD (Tartaglione et al., 2019; Chaliha et al., 2020; Bambini-Junior et al., 2011). To investigate the therapeutic potential of oxytocin for ASD and elucidate its underlying mechanisms, we conducted a comparative analysis of amygdala transcriptome profiles before and after oxytocin administration in an VPA-induced rat model of autism.

## 2 Materials and methods

### 2.1 Materials

#### 2.1.1 Chemicals

Valproic acid sodium salt (P4543-10G) was purchased from Sigma-Aldrich Co. (St Louis, MO, USA). Oxytocin (HY-17571) was purchased from MCE (MedChemExpress LLC, USA).

#### 2.1.2 Animals

Male and female Wistar rats weighing 270–290 g were obtained from the Department of Experimental Animal Center of Guizhou Medical University. Animals were housed individually with water and chow freely available under a regulated environment (23 ± 2°C; 50% ± 10% humidity) with a 12/12 h light-dark cycle. All experiments were approved by Guizhou Medical University Animal Care and Use Committee and conducted in accordance with the National Institutes of Health Guidelines for the Care and Use of Laboratory Animals and with the ARRIVE guidelines.

### 2.2 Methods

#### 2.2.1 Animal model

**Figure 1:**
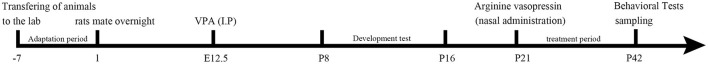
Schematic representation of the experimental procedure.

As previously described (Schneider and Przewlocki, 2005; Dai et al., 2018), female and male rats were allowed to mate overnight at a ratio of 1:1. The morning on which a vaginal plug was found was designated as embryonic day 0.5 (E0.5). The pregnant rats were randomly distributed into two groups: VPA group (*n* = 10) and saline group (*n* = 5). On E12.5, the VPA group were intraperitoneally injected with sodium valproate (dose of 600 mg/kg, 250 mg/ml dissolved in physiological saline); the saline groups were received the same volume of normal saline at same time. The day of birth of the offspring was marked as postnatal day 1 (PND 1). Following weaning on postnatal day 21 (PND 21), the offspring of saline groups were marked as control group, the VPA offspring were randomly divided into two groups: VPA groups (*n* = 10) and VPA+OT group (*n* = 10). The VPA+OT group received a daily intranasal of OT (400 μg/kg, dissolved in physiological saline at a concentration of 2.5 mg/ml) from PND21 to PND42. The control group and the VPA group were given the same amount of physiological saline to control for the effects of injection manipulation. All Experiments were carried out on male offspring. The experimental procedure is shown in Figure 1.

#### 2.2.2 Behavioral tests

##### 2.2.2.1 Open field test

The open field test was used to test for anxiety-like behavior in rats (Kraeuter et al., 2019). The rat was placed in the center of a squared open field box (80 cm L × 80 cm W × 40 cm H) and allowed to explore freely for 5 min. Automatic detection and recorded the locomotion trajectory of rat and calculate the percentage of the time spent in the central zone in 5 min.

##### 2.2.2.2 Three chamber test

The three-chamber test was used to assess the social preference and social novelty of rat by measuring their interaction time with different objects, reflecting their interest in social subjects (Rein et al., 2020). A polyvinyl chloride box was divided in three compartments (each chamber is 40 cm L × 40 cm W × 40 cm H) and both side compartments contained an empty perforated cup. First, the tested rat was allowed to explore freely the whole setting, with all doors open for 10 min (phase 1, adaptation period). After this habituation period, the rat was restricted in the central compartment, while an unfamiliar rat of the same sex (stranger 1) was placed under one of the cups (sides alternated between each rat). The tested rat was then allowed to explore the whole apparatus for 10 min (phase 2, social preference test). After that, it was restricted to the central compartment while another unfamiliar rat of the same sex (stranger 2) was placed under the other cup. The tested rat could then again freely explore the whole apparatus for 10 min (phase 3, social novelty test). In all three phases, time spent in each compartment were manually recorded. The social preferences of the rats were assessed by comparing the dwell times in the left and right compartments.

##### 2.2.2.3 Self-grooming test

To assess repetitive/stereotypic behavior in rats, the self-grooming experiment was used (Mahmood et al., 2020). In brief, animals were individually placed in a plexiglas cage (45 × 25 × 20 cm) and the cumulative grooming duration was measured to quantify stereotyped repetitive behaviors in rats by using stop-watch for 5 min, after a 5 min habituation.

#### 2.2.3 RNA isolation and sequence

The amygdala is the hub of the “social brain”, and dysfunction in this area has been associated with social impairment in individuals with autism (Seguin et al., 2021; Herrero et al., 2020), so we chose to sequence this region. Following decapitation, amygdala tissue (*n* = 5/group) was immediately dissected and frozen in liquid nitrogen for 2 h and then stored at −80°C until further processing. After total RNA was extracted and purified, RNA concentration and purity were assessed quantitatively using a NanoDrop ND-1000 spectrophotometer (NanoDrop, Wilmington, DE, USA), and RNA quality assessed using a Bioanalyzer 2100 system (Agilent, CA, USA). Only RNA samples with an A260/A280 absorbance ratio >1.8 and an RNA Integrity Number (RIN) >7.0 were included in the subsequent analyses. RNA-seq libraries were prepared from the qualified samples and subjected to 2 × 150 bp paired-end sequencing (PE150) on the Illumina Novaseq™ 6000 platform (LC-Bio Technology CO., Ltd., Hangzhou, China).

#### 2.2.4 Bioinformatics analysis for RNA-seq

##### 2.2.4.1 Gene differential expression analysis

Raw data in FASTQ format were generated from the Illumina Novaseq™ 6000. Quality controlled and preprocessed reads were aligned to the reference genome (rattus_norvegicus6.0, v101) using the HISAT2 (Kim et al., 2019), and gene expression quantified using HTSEQ (Anders et al., 2015). Differential gene expression analysis was performed using DESeq2 (Love et al., 2014), with significance thresholds set at fold changes >1.3 and false discovery rate (FDR) < 0.05. Functional enrichment analyses, including Gene Ontology (GO) and KEGG pathway analyses, were conducted using clusterProfiler (Yu et al., 2012).

##### 2.2.4.2 Gene Set Enrichment Analysis

To overcome many genes with moderate but meaningful expression changes are discarded by the strict cutoff value, which leads to a reduction in statistical power, we conduct a Gene Set Enrichment Analysis (GSEA) (Subramanian et al., 2005). GSEA was performed to identify the sets of related genes that might be systematically altered in each group by clusterProfiler (Subramanian et al., 2005; Mootha et al., 2003). The biological process (c5.go.bp.v2024.1.Hs.symbols) and WikiPathways (c2.cp.wikipathways.v2024.1.Hs.symbols) were annotation by Human Molecular Signatures Database v2024 (Liberzon et al., 2015, 2011). Significant gene sets were identified based on |NES| > 1 and NOM *p*-value < 0.05.

##### 2.2.4.3 Gene expression pattern analysis

For expression pattern analysis, soft clustering was performed using time-course sequencing data analysis (TCseq 1.30) with the optimal cluster number determined by default parameters. Subsequently, clusters exhibiting distinct gene expression patterns were subjected to functional annotation analysis using clusterProfiler.

##### 2.2.4.4 Weighted correlation network analysis

WGCNA is a robust analytical method for identifying gene co-expression networks and their associations with clinical phenotypes through scale-free network construction at the transcriptomic level (Langfelder and Horvath, 2008; Zhang et al., 2023). Firstly, the soft threshold power was estimated using nearly scale-free topology to construct a scale-free network. The distance between each gene pair was identified in accordance with the toplogical overlap matrix similarity, followed by hierarchical clustering to identify gene modules, with each branch in the cluster tree representing a distinct module. Modules with eigengene correlations >0.75 were merged to account for expression profile similarity. Module-trait associations were evaluated through Pearson's correlation analysis between module eigengenes (MEs) and clinical traits, with the most significant module (based on module significance, MS) selected for downstream analysis. All analyses were implemented using the WGCNA R package.

#### 2.2.5 qPCR confirmation

Amygdala RNA was extracted using TRIzol (Invitrogen, Carlsbad, CA, United States) and reverse transcribed to cDNA using the Thermo Scientific RevertAid First Strand cDNA Synthesis Kit (Thermo Fisher Scientific Inc., MA, United States). Relative expression level of target mRNA was normalized to GAPDH, and relative expression ratio of a target gene was calculated using the mRNA by the 2^−ΔΔ*CT*^ method (Livak and Schmittgen, 2001). The primer sequences used are listed in Table 1.

**Table 1:** The primer sequences list.

**Gene**	**Primer**	**Sequence (5^′^-3^′^)**	**PCR products**
Rat GAPDH (Zhou et al., 2022)	Forward	ACAGCAACAGGGTGGTGGAC	253bp
	Reverse	TTTGAGGGTGCAGCGAACTT	
Rat Olig2 (Zhou et al., 2022)	Forward	TGAAGAGACTGGTGAGCGAG	165bp
	Reverse	GAGGGAGGATGGGGTGATG	
Rat Cnp (Bao et al., 2024)	Forward	AGCTGCAGTTCCCTTTCCTTCA	274bp
	Reverse	TCATCGAGCACAAGAACCCTGA	
Rat MBP (Zhou et al., 2022)	Forward	ATGTGTTTGGGGAGGCAGAT	233bp
	Reverse	TTGGATGGTCTGAAGCTCGT	

#### 2.2.6 Transmission electron microscopy

Rats (*n* = 2 per group) were perfused transcardially with 2% glutaraldehyde and 2% paraformaldehyde in 0.1 M sodium cacodylate buffer (pH 7.2). The amygdala was dissected out, pre-fixed in 3% glutaraldehyde, post-fixed in 1% osmium tetroxide, dehydrated through a graded series of acetone, embedded in Ep812, semithin sections stained with toluidine blue for optical localization, and ultrathin sections cut with a diamond knife and stained with uranyl acetate and lead citrate for observation by JEM-1400FLASH transmission electron microscopy. The experiments were carried out by the Suzhou PANOMIX Biotech Co, LTD.

### 2.3 Statistical analysis

All data are represented as the mean ± SD. Inter-group statistical significance was determined by Student's *t*-test or one-way ANOVA using STATA 14.0 (StataCorp, College Station, TX, USA). Statistical significance was set at *p* < 0.05.

## 3 Results

### 3.1 Oxytocin rescues prenatal VPA-induced autistic-like behavior in offspring

To validate the reliability of the model and evaluate the efficacy of oxytocin, we compared the behavior change between each group.

#### 3.1.1 Oxytocin rescues prenatal VPA-induced anxiety-like phenotypes in offspring

**Figure 2:**
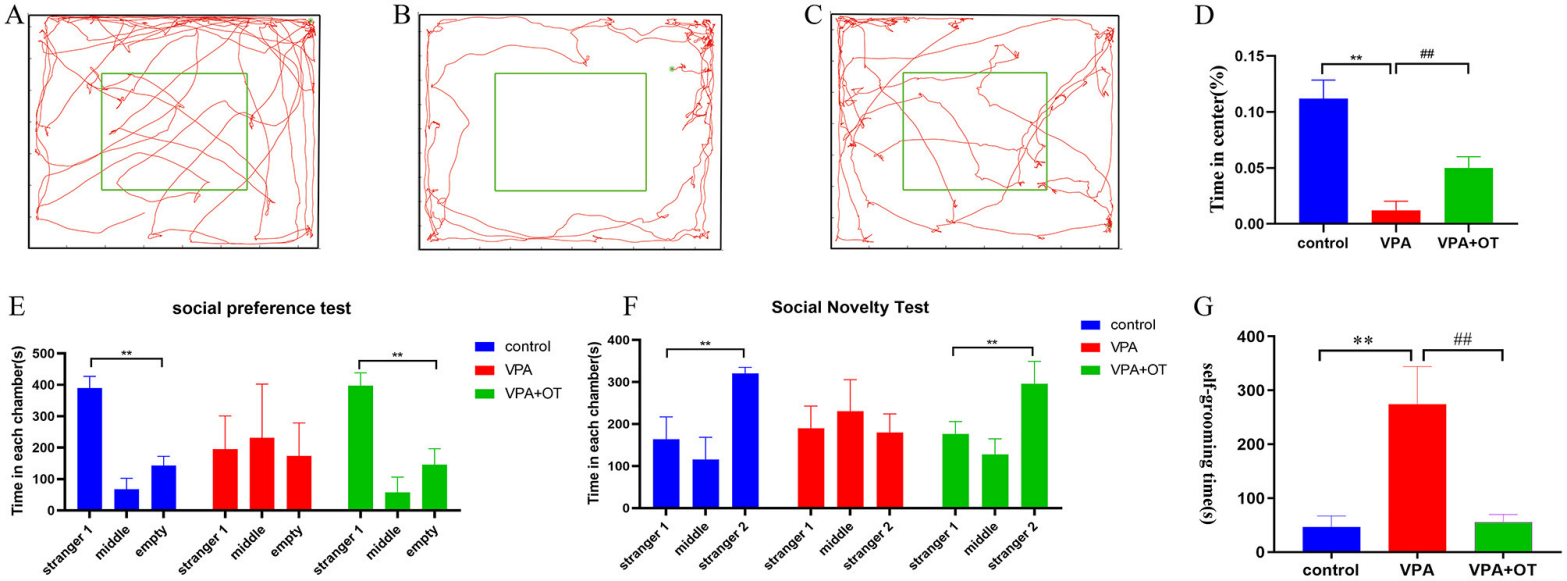
Autistic-like behavior test (*n* = 5 in each group). Locomotion trajectory of **(A)** control group, **(B)** VPA group and **(C)** VPA +OT group, **(D)** Compare the time spend in center in each group. **(E)** Sociability test in each group, **(F)** Social novelty test in each group, **(G)** Repetitive/stereotypic behavior test. Compared with control group: **p < 0.01; compared with VPA-induced autism model group: ^##^*p* < 0.01.

Anxiety-like behaviors were assessed quantitatively using a standardized open field test protocol. Compared to the control group, VPA-exposed rats spent more time on the edges and in the corners of the arena and less time in the central zone (*p* < 0.01). Oxytocin treatment significantly restored the time exploration in center (*p* < 0.01, Figures 2A–D). The results suggest that oxytocin improves the anxiety-like phenotype caused by prenatal VPA in offspring.

#### 3.1.2 Oxytocin rescues prenatal VPA-induced social disorder in offspring

The three-chamber social interaction test was performed to evaluate sociability (social preference) and social novelty recognition. In the social preference test, both control and VPA+OT group spent more time in the side with the unfamiliar rat (Stranger 1) compared to the side of empty cage (*p* < 0.01). The time spent in the side with stranger 1 were not significantly different from the time spent in the empty cage in VPA group, Figure 2E. In the social novelty test, the control and VPA+OT group spent more time in the side with Stranger 2 than in the side with Stranger 1 (*p* < 0.01). The VPAgroup rats spending a comparable time exploring the cage containing stranger 1 and stranger 2 (p > 0.01, Figure 2F). These results suggest that social interaction was impaired in the VPA group and that it was significantly improved following treatment with OT.

#### 3.1.3 self-grooming test

To assess repetitive/stereotypic behavior in rats, the self-grooming experiment was used. Compared to the control group, cumulative self-grooming time was significantly prolonged in the VPA group (*p* < 0.01) and significantly shortened after OT treatment (*p* < 0.01, Figure 2G). The results suggest that oxytocin improves the repetitive/stereotypic phenotype caused by prenatal VPA in offspring.

### 3.2 Changes in the transcriptome before and after oxytocin treatment

Following the confirmation of oxytocin's therapeutic effects through behavioral analyses, we further investigated transcriptomic changes before and after oxytocin treatment to explore its potential mechanisms of action.

#### 3.2.1 Prenatal VPA exposure disrupts key developmental processes in offspring

**Figure 3:**
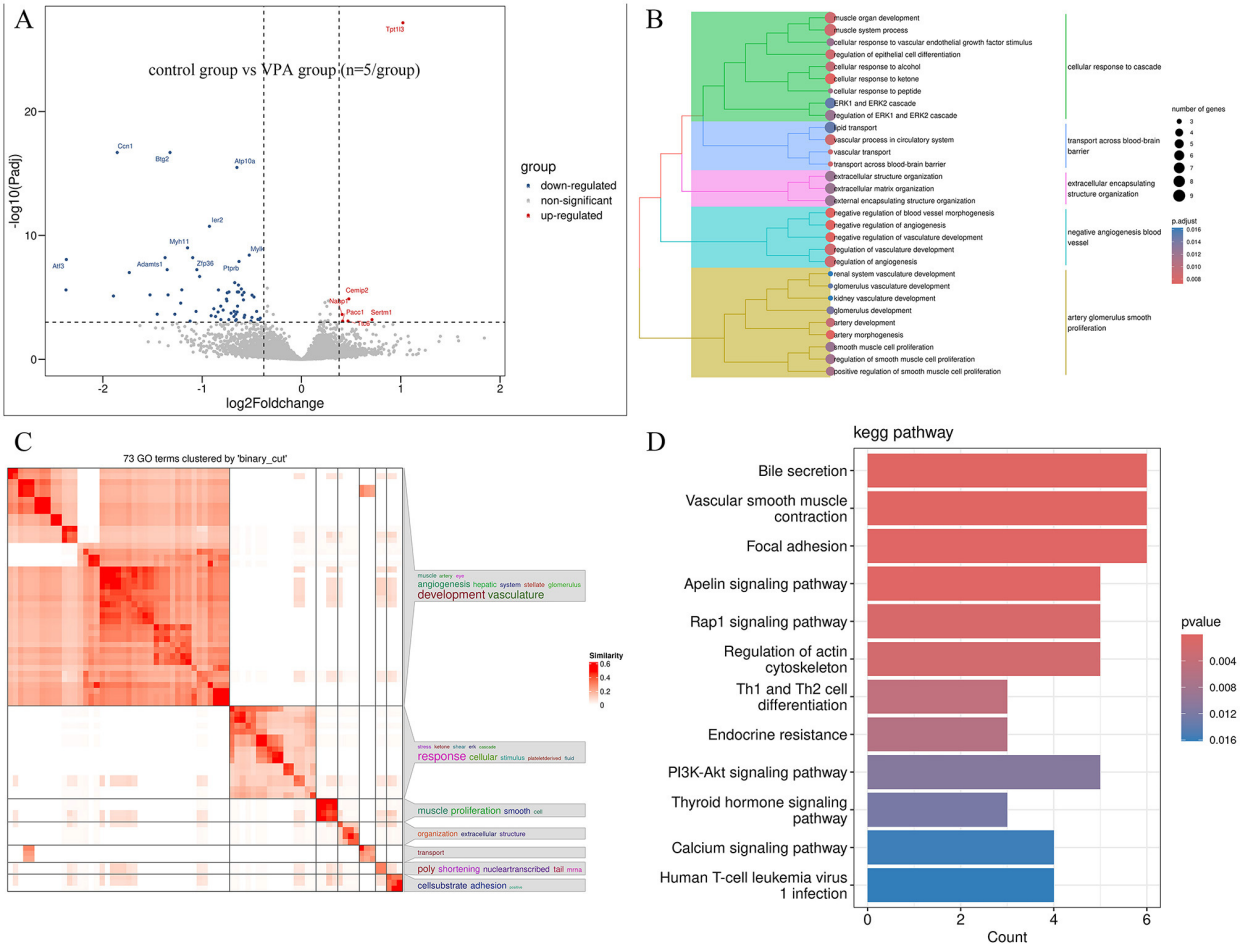
Differentially expressed genes between VPA and Control groups (*n* = 5/group). **(A)** Volcano plot displays differentially expressed genes, **(B)** the results of the GO biology process enriched analysis, **(C)** GO terms clustered by “binary_cut”, **(D)** the results of the KEGG pathway enriched analysis.

There are 71 genes (6 up and 65 down) whose expression was significantly different with an adjusted FDR < 0.05 and | foldchange | ≥ 1.3 in the VPA group compare to control (Figure 3A). GO analyses showed that numerous developmentally relevant biological processes were significantly enriched (Figures 3B, C). Clearly, the VPA group had significant developmental impairments compared with the control group. The KEGG pathway enrichment analysis showed that these differential expression genes were significantly enriched in developmentally relevant pathways, such as PI3K-Akt signaling pathway, Calcium signaling pathway and Focal adhesion, etc. (Figure 3D). The number of differentially expressed genes (9 genes, 7 up and 2 down) between VPAgroup and VPA+OT group was limited and too few for confident GO and KEGG annotation. Given the limited number of differentially expressed genes identified through threshold-based analysis, we will subsequently perform threshold-free enrichment analysis to comprehensively explore potential biological pathways.

#### 3.2.2 Oxytocin significantly ameliorates prenatal VPA-induced impairments in oligodendrocyte development and mitochondrial function in offspring by GSEA

**Figure 4:**
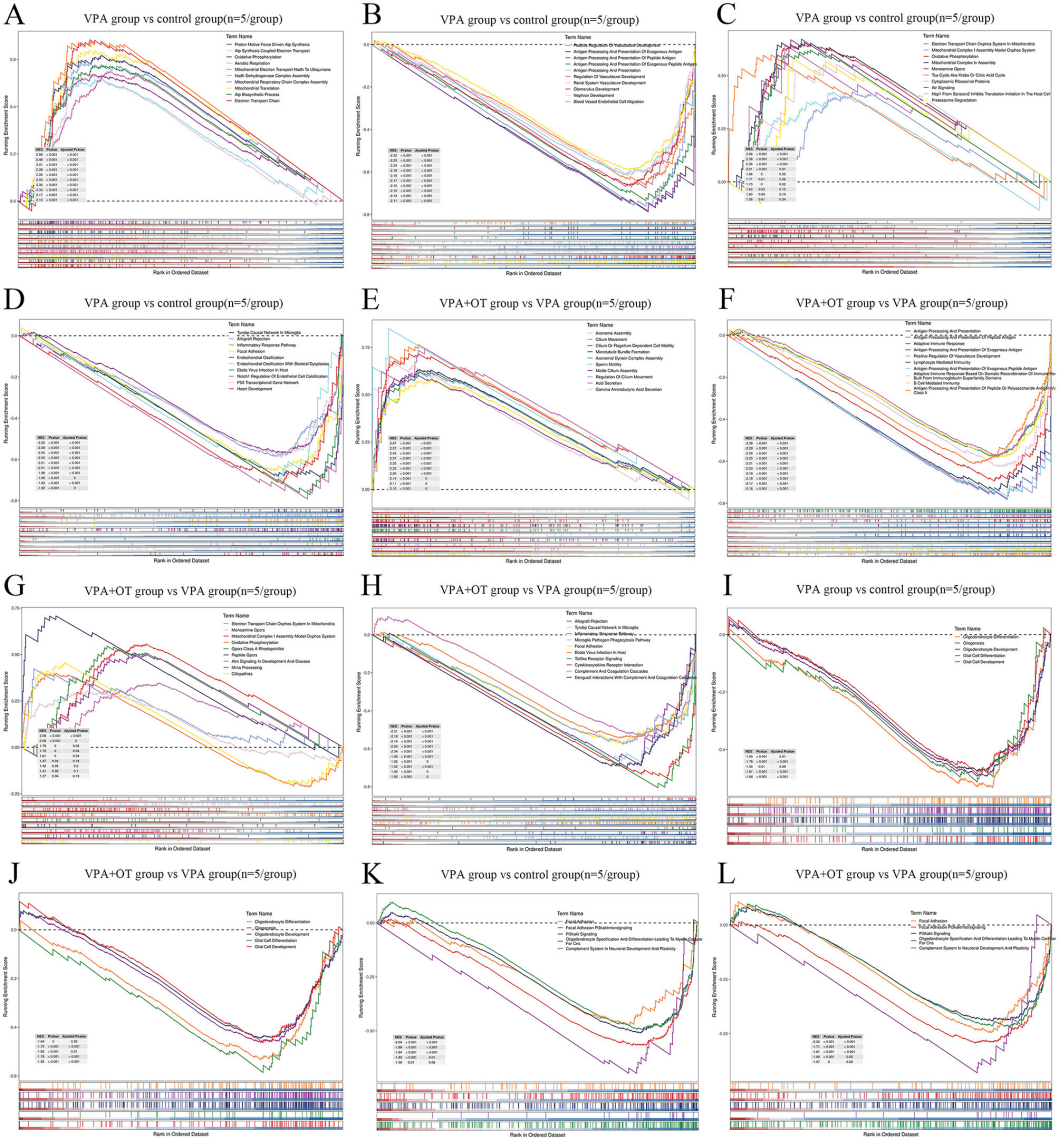
The enrichment plots of GSEA (*n* = 5/group). Compare to control group, **(A)** the top GO biological processes upregulated in VPA group, **(B)** the top GO biological processes downregulated in VPA group, **(C)** the top wiki pathways upregulated in VPA group **(D)** the top wiki pathways downregulated in VPA group. Compare to VPA group, **(E)** the top GO biological processes upregulated in VPA+OT group, **(F)** the top GO biological processes downregulated in VPA+OT group, **(G)** the top wiki pathways upregulated in VPA+OT group, **(H)** the top wiki pathways downregulated in VPA+OT group, **(I)** Glial cell development and differentiation associated biological processes downregulated in VPA group, **(J)** upregulated in VPA+OT group, **(K)** PI3K/AKT signaling pathways downregulated in VPA group, and **(L)** upregulated in VPA+OT group. The significantly enriched gene set were selected with |NES| > 1 and with NOM *p*-value < 0.05.

From a biological perspective, GSEA methods are promising because functionally related genes often display a coordinated expression to accomplish their roles in the cell. Compared to the control group, the results of GSEA GO BP enrichment analysis revealed that biological processes related to mitochondrial energy metabolism, such as proton motive force driven ATP synthesis, ATP synthesis coupled electron transport, and oxidative phosphorylation, were significantly upregulated in VPA group (nom *p* < 0.05, |NES| > 1, Figure 4A). The biological processes associated with development and antigen processing and presentation were significantly downregulated in VPA group (nom *p* < 0.05, |NES| > 1, Figure 4B). The results of GSEA wiki pathway enrichment analysis showed that pathway related to mitochondrial energy metabolism (electron transport chain oxphos system in mitochondria, mitochondrial complex I assembly model oxphos system and oxidative phosphorylation) are significantly upregulated in VPA group (nom *p* < 0.05, |NES| > 1, Figure 4C). TYROBP causal network in microglia, inflammatory response pathway, focal adhesion and so on were significantly downregulated in VPA group (nom *p* < 0.05, |NES| > 1, Figure 4D).

Compare to VPA group, the results of GSEA GO enrichment analysis showed that immune related biological processes (antigen processing and presentation, antigen processing and presentation of peptide antigen, adaptive immune response and so on) are significantly upregulated in VPA+OT group (nom *p* < 0.05, |NES| > 1, Figure 4E). Cytoskeleton related biological processes (axoneme assembly, cilium movement, cilium or flagellum dependent cell motility, among others) are significantly downregulated in VPA+OT group (nom *p* < 0.05, |NES| > 1, Figure 4F). The results of GSEA wiki pathway enrichment analysis showed that TYROBP causal network in microglia, inflammatory response pathway, focal adhesion, among others were significantly upregulated in VPA+OT treatment group (nom *p* < 0.05, |NES| > 1, Figure 4G). Pathway related to mitochondrial energy metabolism (electron transport chain oxphos system in mitochondria, mitochondrial complex I assembly model oxphos system and oxidative phosphorylation, among others) were significantly downregulated in VPA+OT treatment group (nom *p* < 0.05, |NES| > 1, Figure 4H).

The intersection of gene expression variation between down in VPA group (control group vs. VPA group) and up in VPA+OT (VPA group vs. VPA+OT group) were analyzed by Evenn (http://www.ehbio.com/test/venn/#/). The results showed that 335 GO bp were remarkably down regulated in VPA group and up regulated in VPA+OT treatment, such as oligodendrocyte differentiation, glial cell differentiation and development, and gliogenesis, among others (Figure 4I). Twenty-four GO bp were remarkably up-regulated in VPA group and down-regulated in VPA+OT group, such as mitochondrial gene expression, electron transport chain and oxidative phosphorylation and so on (Figure 4J). Fifty-four WP pathways were remarkably down-regulated in the VPA group and up-regulated in the VPA+OT treatment group, such as focal adhesion, focal adhesion PI3K/AKT/MTOR signaling, PI3K/AKT signaling, oligodendrocyte specification and differentiation leading to myelin components for CNS and complement system in neuronal development and plasticity, among others (Figure 4K). Only 3 WP pathways were remarkably up-regulated in the VPA group and down-regulated in VPA+OT group, such as monoamine GPCRs, mitochondrial complex I assembly model oxphos system, and electron transport chain oxphos system in system in mitochondria (Figure 4L).

#### 3.2.3 Oxytocin significantly ameliorates prenatal VPA -induced oligodendrocyte impairments in offspring by dynamic expression pattern analysis

**Figure 5:**
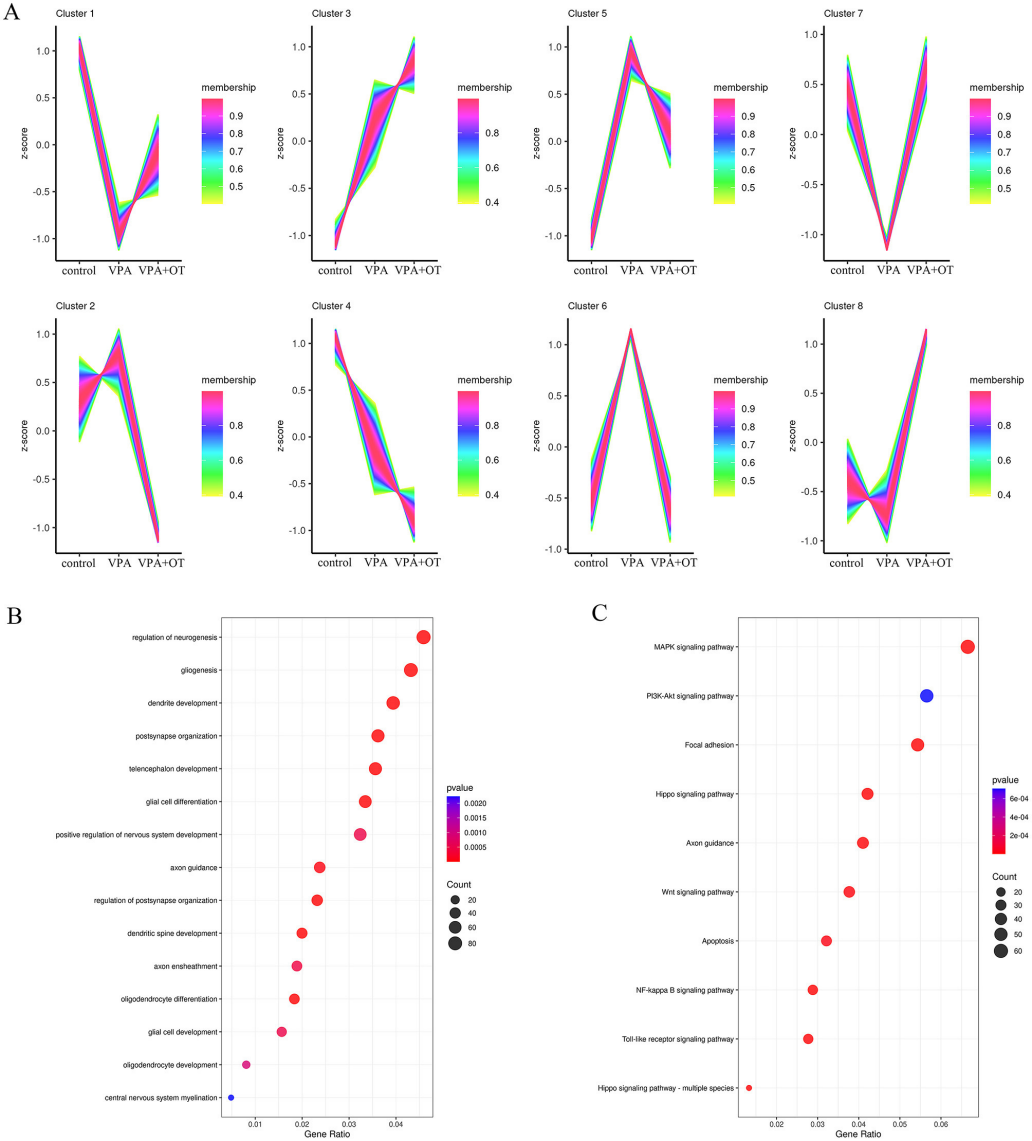
Gene expression pattern cluster (*n* = 5/group). **(A)** Cluster analysis of gene expression patterns among the control, VPA and VPA+OT groups, **(B)** GO of biology process analysis for genes in cluster 7, **(C)** KEGG enriched analysis for genes in cluster 7.

The expression trends of genes at each group were analyzed by TCseq clustering analysis, and 8 clusters with different change trends were screened out (Figure 5A). Based on the above study, we further analyzed the biological functions of genes in cluster 7, which showed a trend of decrease in the VPA group and a significant increase in the VPA+OT group. A large number of development-related biological processes are enriched in cluster 7, such as neurogenesis, gliogenesis, glial cell differentiation and axon guidance and so on (Figure 5B), and these gene were mainly involved in Focal adhesion pathway, PI3K-AKT signaling pathway and MAPK signaling pathway and so on (Figure 5C). The results of expression trends analysis were basically consistent with those of analysis of GSEA.

#### 3.2.4 WGCNA identifies oligodendrocyte-associated module negatively correlated with prenatal VPA-induced phenotypes in offspring

**Figure 6:**
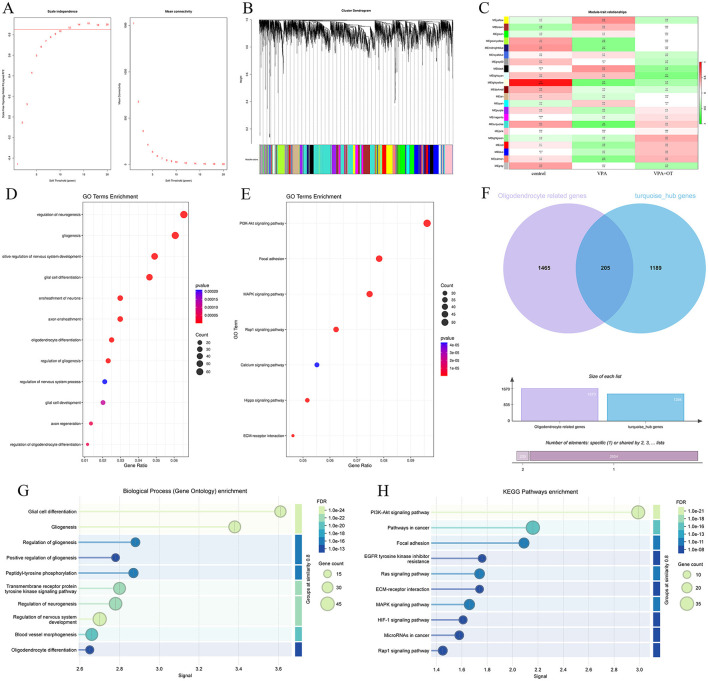
The results of weighted gene co-expression network analysis (*n* = 5/group). **(A)** Scale-free fitting index analysis and mean connectivity of soft threshold power from 1 to 20. **(B)** Dendrogram of all genes was clustered on the basis of a topological overlap matrix. Each branch in the clustering tree represents a gene, while co-expression modules were constructed in different colors. **(C)** Module-trait heatmap of the correlation between the clustering gene module and autism phenotype. **(D)** GO of biological processes annotations for genes in the turquoise module. **(E)** KEGG pathway analysis for genes in the turquoise module. **(F)** Venn diagram showing genes related to oligodendrocyte development and differentiation in turquoise module. **(G)** GO of biological processes annotations and **(H)** KEGG pathway analysis of 205 oligodendrocyte development and differentiation genes in turquoise module.

The top 5,000 most variable genes were selected to construct the co-expression network, and the optimal soft threshold power β of 12 was selected to ensure the scale-free topology model (Figure 6A). Figure 6B shows the gene cluster dendrogram, where each leaf and branch on the tree represents a gene and co-expression module, respectively. Apart from the gray module, we obtained 21 modules, among which the turquoise module, the largest module (Figure 6C), showed a significant negative correlation with autism phenotypes (correlation value = −0.6; *p* = 0.02). Although the light-yellow module also exhibited a significant negative correlation with autism phenotypes (correlation value = −0.54; *p* = 0.04), it was not considered in subsequent analyses due to the relatively small number of genes within the module (46 genes) and its lower correlation and significance compared to the turquoise module. There were no significant positive correlations module in autism phenotype. A total of 1,131 correlation genes were screened from the turquoise module for subsequent analysis. The main biological function of the gene in the turquoise module is to affect the regulation of neurogenesis, gliogenesis, positive regulation of nervous system development, glial cell differentiation, ensheathment of neurons, axon ensheathment, etc. (Figure 6D). These genes primarily affected a series of pathways, such as PI3K-Akt signaling pathway, Focal adhesion, MAPK signaling pathway, Rap1 signaling pathway and so on (Figure 6E). We further extracted 1,465 oligodendrocyte development- and differentiation-related genes from GeneCards (https://www.genecards.org/) and intersected them with genes in the turquoise module, identifying 205 overlapping genes associated with oligodendrocyte development and differentiation (Figure 6F). All of these genes are primarily involved in glial cell differentiation, gliogenesis and regulation of nervous system development (Figure 6G), as well as the PI3K-Akt signaling pathway (Figure 6H).

#### 3.2.5 Oxytocin ameliorates prenatal VPA-induced ultrastructural abnormalities in offspring

**Figure 7:**
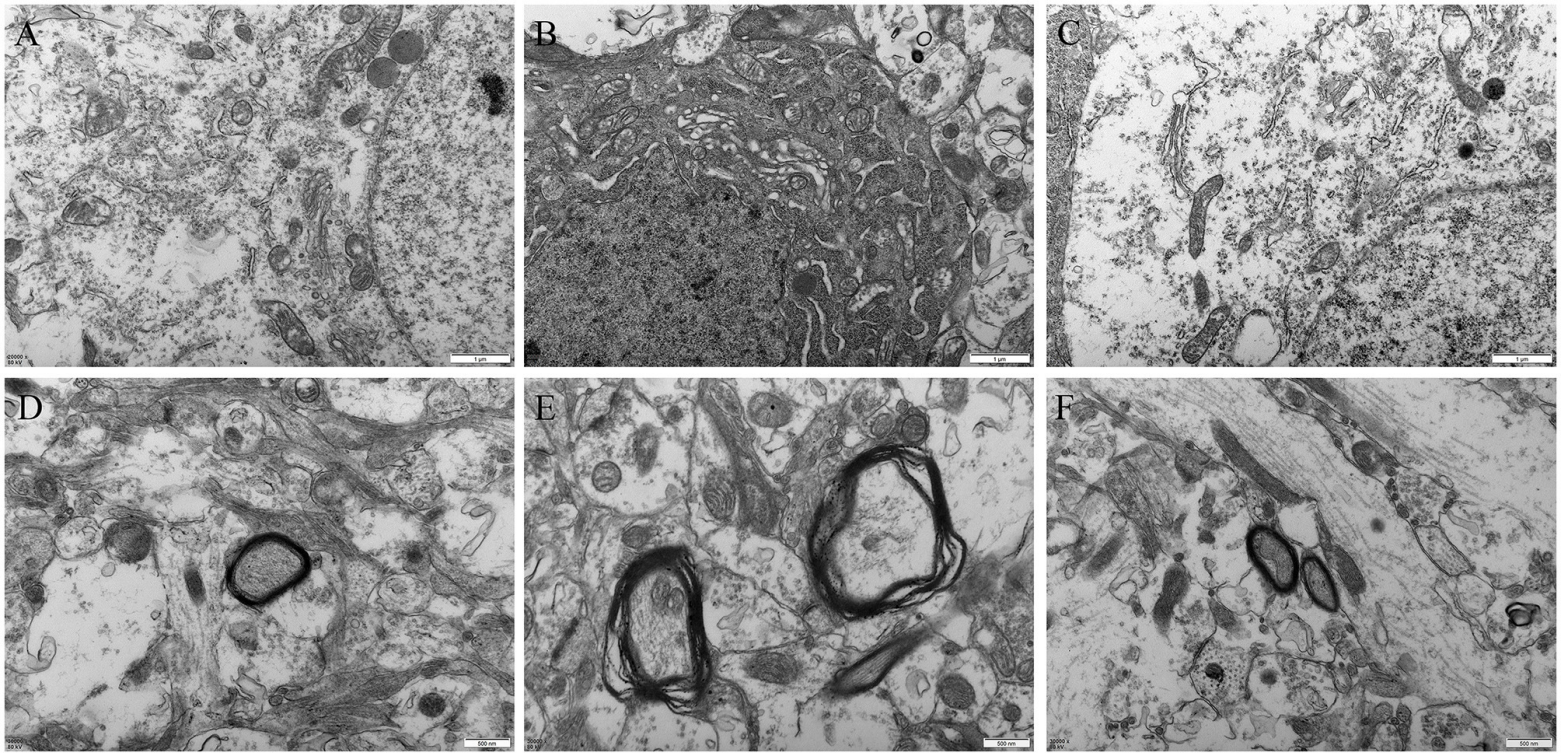
Amygdala ultrastructure comparison between groups (*n* = 2/group). **(A)** neuron in control group, **(B)** neuron in autism VPA group, **(C)** neuron in VPA+OT treatment group**, (D)** myelin sheath in control group, **(E)** myelin sheath in VPA group, **(F)** myelin sheath in VPA+OT treatment group.

Compared with the control group (Figures 7A, D) and VPA+OT treatment group (Figures 7C, F), the VPA group showed obvious mitochondrial swelling and deformation, broken or lost cristae structure, as well as rough endoplasmic reticulum expansion, ribosome shedding and other manifestations of oxidative stress in neurons (Figure 7B). In addition, severe dysmyelination was observed in the VPA group, which characterized by abnormal splitting of myelin lamellae and loss of the regular concentric arrangement (Figure 7E).

#### 3.2.6 Oxytocin treatment rescues the downregulation of oligodendrocyte-related genes in prenatal VPA-exposed offspring

**Figure 8:**
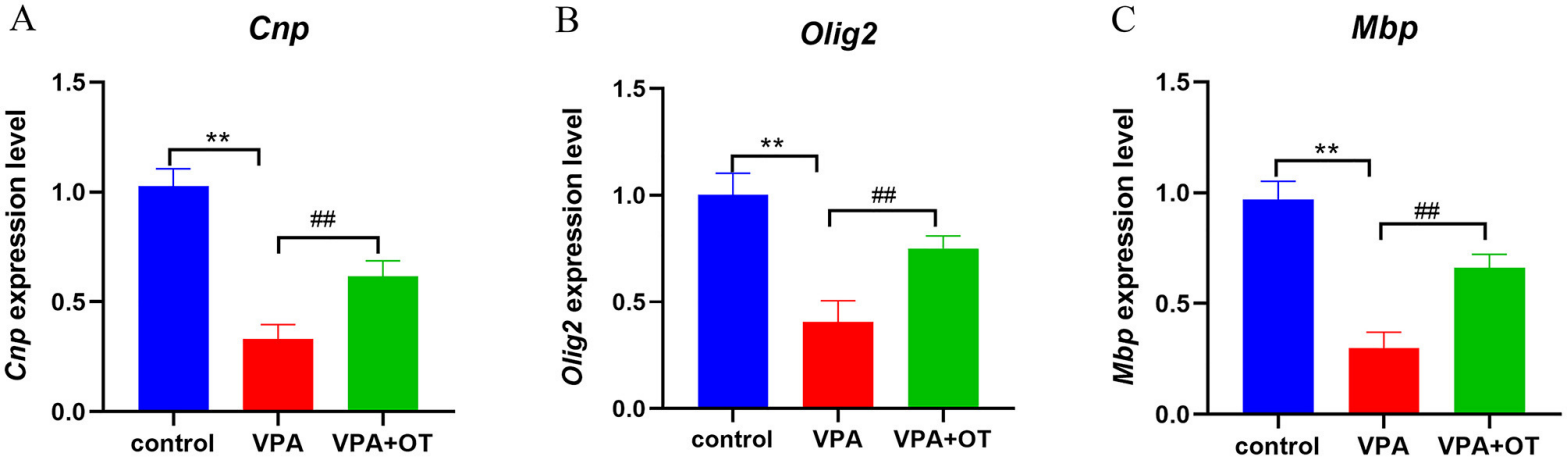
The mRNA levels of oligodendrocyte and myelin development related genes (*n* = 5/group). Compared with control group: **p < 0.01; compared with VPA-induced autism model group: ^##^*p* < 0.01.

Compared with the control group, the mRNA levels of oligodendrocyte and myelin development related genes, such as *Cnp* (a myelin biogenesis related proteins expressed explicitly in differentiating oligodendrocytes in the central nervous system), *Olig2* (a transcription factors necessary for oligodendrocyte development) and *Mbp* (a structural component of myelin, expressed exclusively by myelinating glia) were significantly decreased in VPA group (*p* < 0.01), and the mRNA levels were improved after OT treatment (*p* < 0.01, Figure 8).

## 4 Discussion

### 4.1 Oxytocin improves the ASD-like phenotype by regulating oligodendrocyte development

In the current study, we found that postnatal intranasal oxytocin significantly improved core symptoms of autism (social interaction and stereotypic behaviors) in an offspring model of prenatal valproate exposure rats.

To further elucidate the underlying mechanisms, we performed a comprehensive comparative analysis of amygdala gene expression profiles before and after OT intervention.

By combining transcriptome analysis with advanced bioinformatics methods, we have consistently shown that biological processes associated with oligodendrocyte development and differentiation are significantly inhibited in the amygdala of a rat model of autism. Notably, administration of oxytocin was effective in ameliorating these impaired biological processes. These findings were subsequently validated through qPCR analysis and ultrastructural examination using transmission electron microscopy, providing robust evidence for the therapeutic potential of OT in modulating oligodendrocyte development in autism spectrum disorder. The results were consistent with the research of (Dalton et al. 2024); (Zhou et al. 2022); and Zhang X. F. et al. (2020) who found the transcript level of myelin-related genes were reduce in autism model. Obviously, deficits in OL development and function may be one of the core mechanisms of autism. Abnormal development of oligodendrocytes (OLs) and their precursor cells (OPCs) can disrupt myelin formation, metabolic support and immune regulation, severely impairing neural circuit function and causing social interaction deficits in autism (Galvez-Contreras et al., 2020; Kuhn et al., 2019; Phan et al., 2020; Simons and Nave, 2015; Saab et al., 2013; Xiao and Czopka, 2023; Steinman, 2020; Cho et al., 2023). In addition, we also found that biological processes related to mitochondrial function, such as mitochondrial gene expression, the electron transport chain and oxidative phosphorylation, were over-activated in the autism model and improved after OT treatment. Overactivation of oxidative phosphorylation and the electron transport chain is typically accompanied by excessive generation of reactive oxygen species (ROS) and subsequent oxidative stress (Bjørklund et al., 2020). These molecular alterations were consistently reflected in our transmission electron microscopy analyses, which revealed characteristic ultrastructural abnormalities, mitochondrial swelling and deformation, broken or lost cristae structure, as well as rough endoplasmic reticulum expansion. Dysfunction of the mitochondrial electron transport chain and oxidative phosphorylation has been demonstrated in autism (Frye et al., 2024; Gevezova et al., 2020; Citrigno et al., 2020; Mahalaxmi et al., 2021), but whether this is enhanced (Hassan et al., 2021; Park et al., 2018; Frye and Naviaux, 2011; Palmieri et al., 2010) or attenuated remains controversial (Chauhan et al., 2011; Chen et al., 2025; Wang et al., 2022). (Matsuo et al. 2020) discovered that prenatal exposure to VPA induces mitochondrial dysfunction, characterized by reduced activities of electron transport chain complexes I and II, elevated complex IV activity, and decreased ATP production, all of which were significantly ameliorated by oxytocin treatment. (Kumar et al. 2015) found that exposure to valproic acid during pregnancy resulted in decreased activity of mitochondrial complexes I, II, and IV in the prefrontal cortex, as well as enhanced oxidative stress. Pathway enrichment analysis revealed significant dysregulation of the PI3K/Akt pathway in the VPA group, which was subsequently ameliorated by OT treatment. This phenomenon may be attributed to inhibition of the PI3K signaling pathway can lead to up-regulation of DNA methyltransferases (DNMT1 and DNMT3A), which repress the expression of genes involved in neural differentiation and maturation. Conversely, PI3K activation promotes the upregulation of DNA demethylation enzymes, thereby reactivating neurogenesis-associated genes and facilitating neurodevelopmental processes (Baranova et al., 2021). However, the activity of the PI3K/Akt pathway in autism is not entirely consistent, with (Nicolini et al. 2015); (Yadollahi-Farsani et al. 2024); and Wang L. et al. (2024) confirming our findings that PI3K activity is significantly reduced in ASD, while (Thomas et al. 2023); (Sharma et al. 2022); (Li et al. 2024); and Zhang W. et al. (2020) found the opposite. The inconsistent results may be due to the sample size, different brain regions, different developmental time and different research methods, among others.

## 5 Limitations

Our study has several limitations. First, we only tested in male offspring. We did this for two main reasons (1) Significant gender differences have been observed in patients with clinical ASD, with a notably higher prevalence among males than females (~4:1 male-to-female ratio) (2) Male animals are more susceptible to VPA-induced ASD than females (Gouda et al., 2022; Zarate-Lopez et al., 2024; Kazlauskas et al., 2019). Androgen hormones may be the main reason for this difference. Some researchers believe that testosterone may interact with downstream molecules such as neurotransmitters, neuropeptides, and immune pathways, thereby contributing to male vulnerability (Knickmeyer and Baron-Cohen, 2006; Wang Z. et al., 2024). By contrast, estrogens may promote social recognition (Jiang et al., 2025), possibly by inhibiting oxidative stress, reducing neuroinflammation and regulating oxytocin production in the hypothalamus and OT receptors in the medial amygdala (Gabor et al., 2012). Secondly, although the potential effects of oxytocin on control animals were not examined directly in this study, the existing literature clearly shows that the administration of oxytocin increases prosocial behavior in neurotypical rodents and humans (Froemke and Young, 2021; Bauman et al., 2018). This lends biological plausibility to our intervention design. Future studies could explicitly compare oxytocin responses between ASD models and control subjects to delineate disease-specific mechanisms. Thirdly, our transcriptome analyses revealed a low number of differential genes, particularly when comparing the VPA group with the VPA+OT group. A persistent challenge in transcriptome studies—particularly in neurodevelopmental disorders like ASD, is the establishment of biologically meaningful thresholds for differential expression analysis. Combined with previous studies (Dalton et al., 2024; Kim et al., 2022; Rexrode et al., 2024; Crittenden et al., 2021; Zhang et al., 2018; Matsuo et al., 2022), we found that conventional bulk transcriptome threshold (FDR < 0.05 and fold change >2) are unsuitable for neuro-omics analyses in autism. This may be related to factors such as small sample size, high sample heterogeneity, and spatio-temporal specific expression of neurological genes, among others (Vaes et al., 2014; Kang et al., 2011; Chou et al., 2016; Naumova et al., 2013). Overly strict thresholds may miss some truly differentially expressed and meaningful genes, especially with small sample sizes. To overcome the shortcomings of the above analyses, we have relaxed cutoffs thresholds (FDR < 0.05 and fold change >1.3) while at the same time applying a variety of threshold-free analyses (GSEA, Dynamic expression patterns and WGCNA) to increase the reliability of the analyses. Our research results demonstrate that in transcriptome sequencing analysis, combining threshold-based methods (e.g., differential expression analysis) with threshold-free approaches (such as GSEA and WGCNA) can reduce false negatives, enhance the reliability of pathway discovery, and yield more robust results.

## 6 Translation challenges

Despite promising preclinical evidence supporting oxytocin's therapeutic potential for ASD, its translation into clinical practice faces substantial challenges. Current evidence indicates that clinical trial outcomes show significant variability, with the efficacy of oxytocin interventions being strongly influenced by both individual patient characteristics (including genetic polymorphisms, developmental stages, sex differences, and clinical subtypes), intervention parameters (such as optimal dosage, administration routes, and treatment duration) (Lefter et al., 2020; Martins et al., 2022; Audunsdottir et al., 2024; Ricchiuti et al., 2025) and the species-specific differences. Exposure to environmental risk factors (VPA, Poly I:C, HTX and GDM, etc.) during pregnancy mirrors the natural progression of autism, reproducing the social and cognitive impairments experienced by individuals with the condition. However, the neurobiological mechanisms behind these models are not entirely consistent. The VPA model primarily affects epigenetic regulation by inhibiting histone deacetylases, leading to abnormal neuronal synaptic plasticity, impaired oligodendrocyte differentiation, and mitochondrial dysfunction. The Poly I:C model mimics maternal immune activation, predominantly inducing Th17-mediated neuroinflammation and microglial overactivation, which consequently disrupts synaptic pruning, myelination, and mitochondrial function (Naviaux et al., 2013). The HTX model focuses on the impact of thyroid hormone deficiency on neurodevelopment, manifesting as dysregulated expression of glutamatergic synaptic proteins and trigger proinflammatory responses (González-Madrid et al., 2024). The GDM model triggers metabolic disturbances through hyperglycemia and insulin resistance, resulting in oxidative stress and chronic inflammation that ultimately cause neuronal excitation-inhibition imbalance and oligodendrocyte precursor cells dysfunction (Liu et al., 2025). Additionally, significant differences in oxytocin systems exist between species during the transformation process. For example, the distribution of oxytocin receptors, the permeability of the blood-brain barrier and the neural circuits underlying social behavior differ between humans and rats (Freeman and Young, 2016).

## 7 Conclusion

Collectively, our results showed that OT significantly improved the differentiation and development of oligodendrocytes, and mitochondrial oxidative phosphorylation in the amygdala of autistic rats, and these neuroprotective effects were likely mediated through PI3K/Akt signaling pathway activation in our autism rat model. This found provides a novel theoretical foundation for oxytocin-based ASD therapies and highlights oligodendrocyte-targeted interventions as a promising therapeutic strategy.

## Data Availability

The original contributions presented in the study are publicly available. This data can be found here: https://github.com/xiaomaozhoubo/Oxytocin-treatment-for-autism.
